# Natural Variation in Volatile Emissions of the Invasive Weed *Calluna vulgaris* in New Zealand

**DOI:** 10.3390/plants9020283

**Published:** 2020-02-21

**Authors:** Evans Effah, D. Paul Barrett, Paul G. Peterson, A. Jonathan R. Godfrey, Murray A. Potter, Jarmo K. Holopainen, Andrea Clavijo McCormick

**Affiliations:** 1 School of Agriculture and Environment, Massey University, Private Bag 11-222, Palmerston North 4442, New Zealand; e.effah@massey.ac.nz (E.E.); d.p.barrett@massey.ac.nz (D.P.B.); m.potter@massey.ac.nz (M.A.P.); 2 Manaaki Whenua-Landcare Research, Riddet Road, Massey University, Palmerston North 4474, New Zealand; petersonp@landcareresearch.co.nz; 3 School of Fundamental Sciences, Massey University, Private Bag 11-222, Palmerston North 4442, New Zealand; a.j.godfrey@massey.ac.nz; 4 Department of Environmental and Biological Sciences, University of Eastern Finland, P.O. Box 1627, FI-70211 Kuopio, Finland; jarmo.holopainen@uef.fi

**Keywords:** heather, invasive species, plant scents, plant secondary metabolites, soil nutrients, volatile organic compounds

## Abstract

Invasive plants pose a threat to natural ecosystems, changing the community composition and ecological dynamics. One aspect that has received little attention is the production and emission of volatile organic compounds (VOCs) by invasive plants. Investigating VOCs is important because they are involved in vital ecological interactions such as pollination, herbivory and plant competition. Heather, *Calluna vulgaris*, is a major invasive weed in New Zealand, especially on the Central Plateau, where it has spread rapidly since its introduction in 1912, outcompeting native species. However, the chemical behaviour of heather in its invaded ranges is poorly understood. We aimed to explore the natural variation in volatile emissions of heather and the biotic and abiotic factors influencing them on the Central Plateau of New Zealand. To this end, foliar volatiles produced by heather at four different sites were collected and analysed using gas chromatography coupled to mass spectrometry. Soil properties, herbivory and other environmental data were also collected at each site to investigate their effects on VOC emissions using generalised linear models (GLMs). Our results reveal significant differences in VOC emissions between sites and suggest that soil nutrients are the main factor accounting for these differences. Herbivory and temperature had only a minor effect, while soil water content had no impact. Further studies are needed to investigate how these variations in the invasive plant’s foliar volatiles influence native species.

## 1. Introduction

The intentional or accidental introduction of exotic species, including plants, into new regions, poses a threat to biodiversity [1]. Plant invasions have escalated in recent times, mainly because of increased human migration, global trade, and climate change [2,3,4]. Many morphological, physiological and reproductive traits associated with invasiveness in plants have been explored to understand and mitigate negative impacts [5]. Chemical research has focused on the allelopathic properties of exudates (fluids) released by invasive plants [6,7,8,9], while much less is known about volatile organic compounds (VOCs)—scents—and their ecological impacts [10]. VOCs are the main currency in plant communication, mediating multiple interactions between the emitting plant and other organisms including beneficial arthropods (such as pollinators and seed dispersers), herbivores, natural enemies of herbivores, microorganisms (e.g., mycorrhizae), pathogens and other plants [11].

Plant volatile emissions are species-specific [12] but also plastic to the changing environment, and are known to change in response to biotic variables such as herbivory and pathogen attack, and abiotic variables such as temperature, soil nutrients, and ultraviolet-B radiation (UV-B), among others [13]. Previous studies suggest that VOCs emitted by invasive species can inhibit seed germination and reduce the above and below-ground growth of nearby plants, with direct benefits to the emitting plant [14,15,16,17]. Due to their ecological value and allelopathic effects, VOCs can be considered valuable “weapons” in plant competition [10,18]. Given the relevance of plant volatiles, the aim of this study is to characterise the natural variation in VOC emissions of an invasive species in the field and explore the biotic and abiotic factors contributing to this variation.

**Figure 1:**
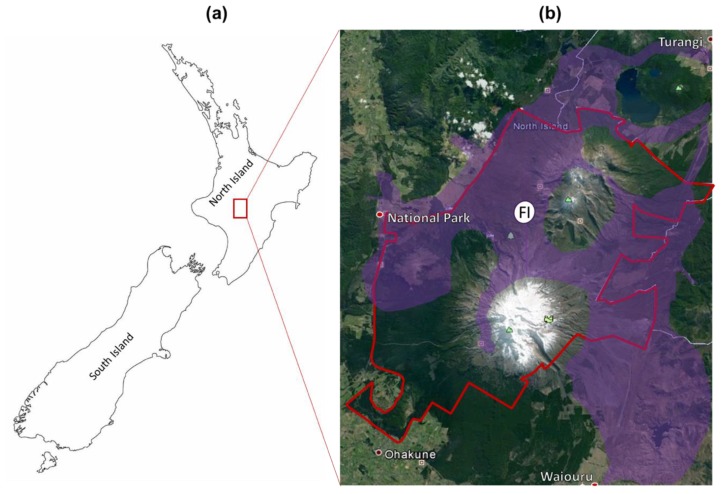
The distribution of heather in the Central North Island. (**a**) North and South Islands of New Zealand. (**b**) Invasion of heather in the Central Plateau. Heather was first planted in the white region (FI) but has now spread through all the regions in purple. The boundary of Tongariro National Park (TNP) is shown in red.

The Central Plateau is a volcanic area covering the central part of the North Island of New Zealand, including the Tongariro Natural Park (TNP), a world heritage site of natural and cultural value. Common native plants in this area include *Chionochloa rubra* (red tussock) and *Poa colensoi* (blue tussock) (both grasses), and *Leptospermum scoparium* (mānuka) and *Dracophyllum subulatum* (*Dracophyllum*) (both woody perennial species) [19]. *Calluna vulgaris* (heather) is a European native perennial shrub from the family Ericaceae. This alien species was deliberately introduced to the Central Plateau in 1912 [20,21] and is now the most widespread invasive weed in this area, covering more than 50,000 ha of the TNP and surrounding land (Figure 1), representing the most substantial infestation of heather in New Zealand.

On the Central Plateau, heather has invaded the seral tussock grasslands, modifying soil properties, outcompeting native vegetation and disrupting the natural processes of plant regeneration and succession [22,23,24,25]. Phytophagous insect diversity and abundance are also negatively affected by heather invasion due to the changing habitat, loss or reduction of native food plants and increased arachnid predation [22]. Efforts to control this species, including the use of herbicides and introduction of a Chrysomelid biocontrol agent *Lochmaea suturalis,* have been of limited success due to the persistence of seedbank in the soil and poor establishment of the biocontrol agent [23,26].

A recent study explored the VOC emissions of heather as a main component of heath ecosystems in its native range (Denmark) [27] and found this species to be a rich terpenoid emitter, reporting at least 15 monoterpenes and the homoterpene (*E*)-4,8-Dimethyl-1,3,7-nonatriene. The authors investigated seasonal variation and emission responses to six years of climatic manipulations (elevated CO_2_, extended summer drought and night-time warming) in a semi-natural setting and found seasonal variation in VOC emission but also a significant effect of the abiotic variables tested. However, to our knowledge, our study is the first to explore the VOC emissions of heather in its invasive range and the factors affecting their emissions under natural conditions (without experimental manipulation).

## 2. Results

### 2.1. Volatile Emissions by Heather

**Figure 2:**
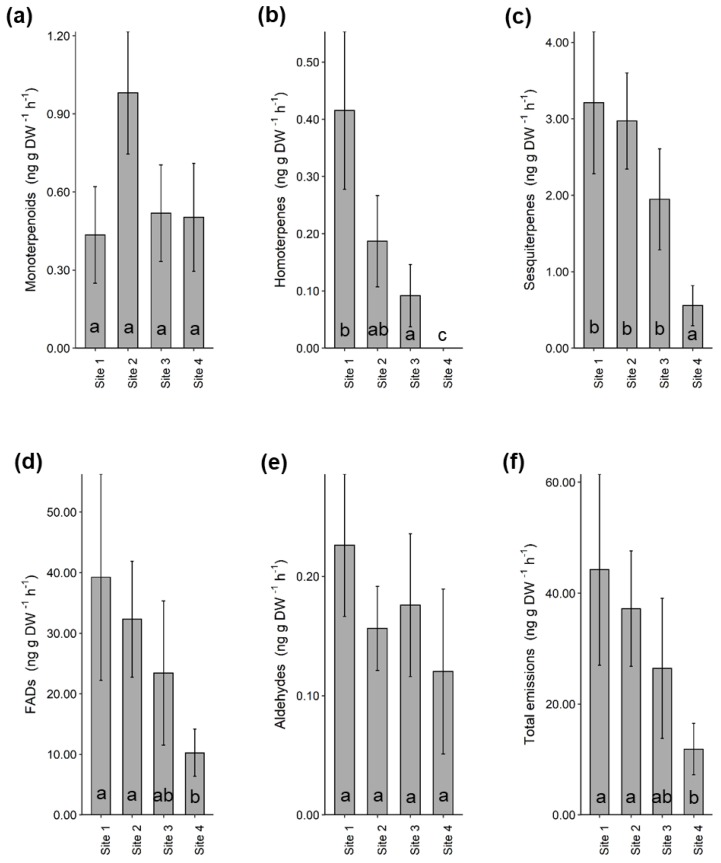
Volatile organic compound (VOC) classes identified from the headspace of heather at four different sites. Bars show mean ± SE of total (**a**) monoterpenoids, (**b**) homoterpenes, (**c**) sesquiterpenes, (**d**) fatty acid derivatives, (**e**) aldehydes and (**f**) total volatile emissions measured from target plants from each site (n = 5). Letters indicate pairwise comparisons between sites. Abbreviations: fatty acid derivatives (FADs).

After collecting samples from the headspace (i.e., surrounding airspace) of heather branches enclosed in nylon cooking bags and analysing them using a Gas Chromatograph-Mass Spectrometer (GC-MS), we identified 33 volatile compounds and grouped them under their respective chemical classes (Table S2). The most abundant compounds were fatty acid derivatives (33.3%), monoterpenes (21.2%) and sesquiterpenes (33.3%). Of the 33 VOCs identified from heather, a typical fungal volatile 1-octen-3-ol [28] was among the most abundant compounds at all sites (Table S2). A comparison between sites revealed that total volatile emissions were significantly lower at site 4 when compared to site 1 (GLM; *β* = −1.32, *X*^2^ = 5.66, *p* = 0.017) and site 2 (GLM; *β* = −1.14, *X*^2^ = 4.27, *p* = 0.039). The same was true for homoterpenes, sesquiterpenes and fatty acid derivatives (Figure 2). Total fatty acid derivatives were significantly lower at site 4 compared to site 1 (GLM; *β* = −1.34, *X*^2^= 5.30, *p* = 0.021) and site 2 (GLM; *β* = −1.15, *X*^2^ = 3.88, *p* = 0.049). Total sesquiterpenes were also significantly lower at site 4 compared to site 1 (GLM; *β* = −1.75, *X*^2^ = 13.20, *p* < 0.001), site 2 (GLM; *β* = −1.68, *X*^2^ = 12.0, *p* = 0.001) and site 3 (GLM; *β* = −1.25, *X^2^* = 6.72, *p* = 0.010). The proportion of homoterpenes was significantly higher at site 1 (GLM; *β* = 6.03, *X*^2^ = 114.30, *p* < 0.001), site 2 (GLM; *β* = 5.24, *X*^2^ = 86.10, *p* < 0.001) and site 3 (GLM; *β* = 4.53, *X*^2^ = 64.50, *p* < 0.001) compared to site 4.

### 2.2. Principal Component Analysis Based on Volatiles Emissions at Different Sites

**Figure 3:**
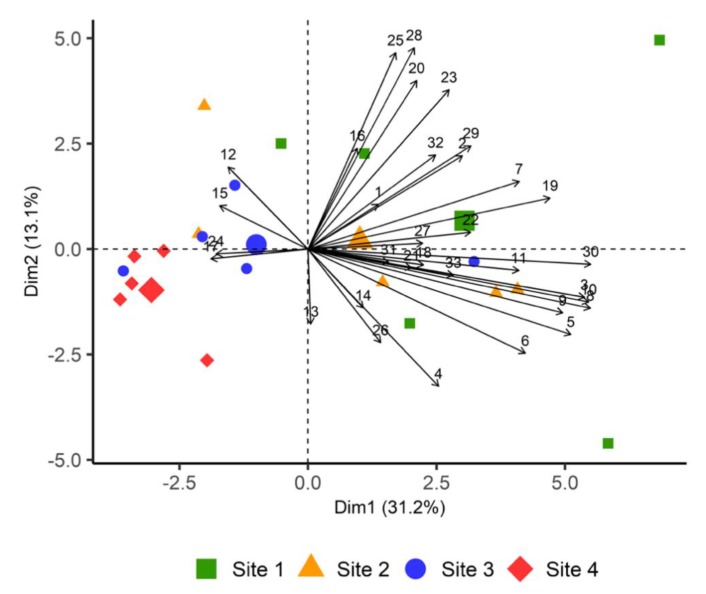
Principal components analysis (PCA) biplot showing PC scores of individuals and loadings of variables. PCA was based on 33 VOCs emitted by heather from all sites. The numbers in the graph indicate the following compounds; (1) hexyl acetate, (2) 1-hexanol, (3) (*Z*)-2-hexenol, (4) (*Z*)-3-hexenol, (5) (*Z*)-3-hexenyl 2-methylbutyrate, (6) (*Z*)-3-hexenyl acetate, (7) (*Z*)-3-hexenyl benzoate, (8) (*Z*)-3-hexenyl butyrate, (9) (*Z*)-3-hexenyl hexanoate, (10) (*Z*)-3-hexenyl isobutyrate, (11) (*Z*)-3-hexenyl valerate, (12) α-pinene, (13) α-terpineol, (14) β-myrcene, (15) β-pinene, (16) limonene, (17) linalool, (18) (*Z*)-β-ocimene, (19) (*E*,*E*)-α-farnesene, (20) α-gurjunene, (21) (*E*)-β-caryophellene, (22) δ-cadinene, (23) γ-elemene, (24) copaene, (25) germacrene B, (26) germacrene D, (27) humulene, (28) (*E*)-β-famesene, (29) (*Z*,*E*)-α-farnesene, (30) (*E*)-4,8-Dimethyl-1,3,7-nonatriene, (31) 1-octen-3-ol, (32) decanal, (33) nonanal. Compounds were assigned to the following classes: fatty acid derivatives (1–11), monoterpenes (12–18), sesquiterpenes (19–29), homoterpene (30), alcohol (31), aldehydes (32–33).

We used principal component analysis (PCA) to further explore differences in plant volatile emission between sites. The first axis of principal component analysis (PC1) explained about 31% of the total variance in VOC emissions among the four sites and was mostly characterized by fatty acid derivatives and (*E*)-4,8-Dimethyl-1,3,7-nonatriene ((*E*)-DMNT) (Figure 3). PC2 was characterized by sesquiterpenes and explained about 13% of the variability. The first six principal components (PC1 to PC 6) captured over 78% of the variability in the data and were considered in subsequent analyses. Sites 1 and 4 were clearly separated from one another based on VOC emitted by heather at these sites, while sites 1, 2 and 3 overlapped.

### 2.3. Soil Nutrients and Environmental Variables

We collected soil samples and environmental data (daytime temperature, soil water content and soil temperature) at each site. Soils analyses revealed that all sites were nutrient poor; however, at site 4, most nutrients, except for N, were lower (Table 1).

**Figure 4:**
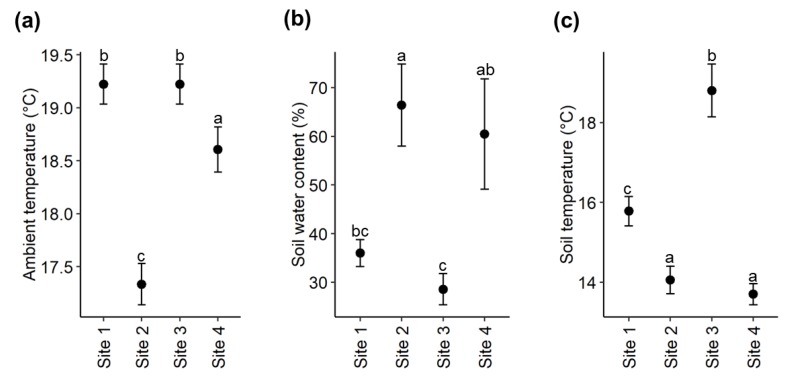
Comparison of (**a**) ambient daytime temperature, (**b**) soil water content and (**c**) soil temperature between sites. The *y*-axis showing mean ± SE values and *x*-axis representing the four study sites. Different letters indicate significant differences.

The ambient daytime temperature differed significantly between the four study sites (Kruskal-Wallis; *X*^2^ = 58.275, *df* = 3, *p* < 0.001), with site 2 having a significantly lower temperature compared to the other three sites (Figure 4a).

The soil water content (SWC) also differed significantly between the four study sites (ANOVA; *F*_3_,_16_ = 6.206, *p* = 0.005, Figure 4b). Site 2 had a higher SWC than site 3 (Tukey’s HSD; *p* = 0.011) and site 1 (Tukey’s HSD; *p* = 0.046).

There was also a significant difference in soil temperature between the four sites (Kruskal–Wallis; *X*^2^ = 15.736, *df* = 3, *p* = 0.001) with site 3 having the highest soil temperature, while the lowest soil temperature was recorded at sites 2 and 4 (Figure 4c).

### 2.4. Arthropod Community Composition

**Figure 5:**
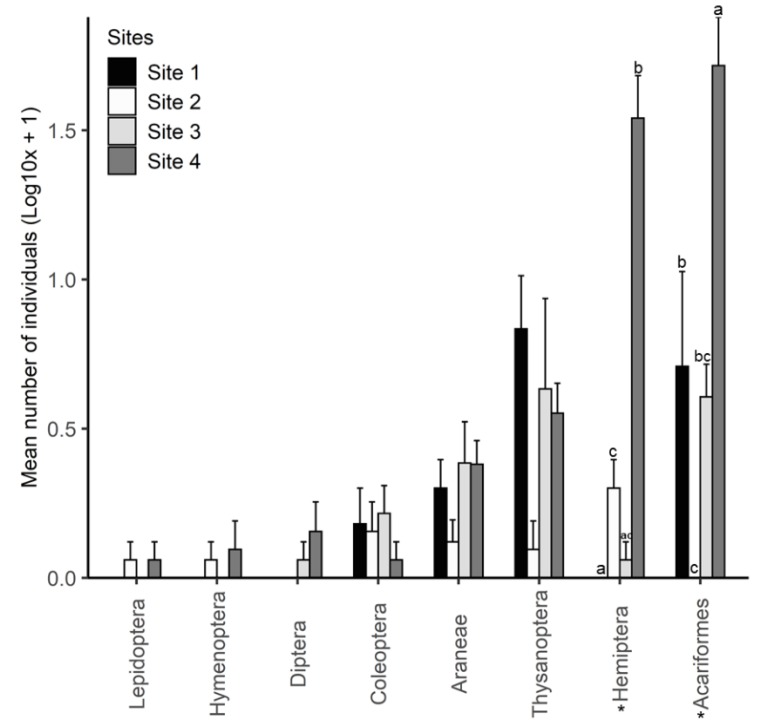
Arthropod community composition from study sites determined by beating a branch of target heather plants on a tray (n = 5). Bars show mean number ± SE of individuals in the respective arthropod orders. Arthropod groups with asterisks (*) were significantly different between sites.

Using a beating tray, arthropods were collected from the sampled heather plants (five per site) with Hemiptera, thrips, spiders and mites being the most dominant groups (Figure 5). The number of Hemiptera was significantly different between the four sites (Kruskal–Wallis; *X*^2^ = 15.697, *df* = 3, *p* = 0.001). There was a greater number of Hemiptera on heather at site 4 in comparison to the other sites (Figure 5). Similarly, the number of mites on heather also differed significantly between the four sites (Kruskal–Wallis; *X*^2^ = 13.012, *df* = 3, *p* = 0.005), with a greater number of mites at site 4. The number of thrips found on heather at site 1 was higher than the other sites, but only marginally significant (Kruskal–Wallis; *X*^2^ = 7.698, *df* = 3, *p* = 0.053, Figure 5).

### 2.5. Herbivore Damage on Heather

**Figure 6:**
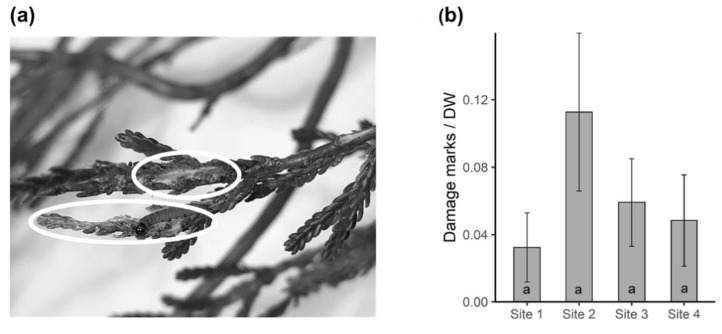
(**a**) Visible herbivore damage on heather. Sections in white circles were counted as two separate damage events. (**b**) Mean number of damage marks counted on target plants (n = 5) for each site expressed as damage per gram dry weight.

Herbivore damage on heather was recorded by counting the number of visible damage marks on the branches used for volatile collection. The distribution of herbivore damage on heather was not significantly different between the four sites (Kruskal–Wallis; *X*^2^ = 2.475, *df* = 3, *p* = 0.480) although slightly higher damage was recorded on the plants at site 2 (Figure 6b).

### 2.6. Effect of Biotic and Abiotic Factors on Volatile Emissions

We used Generalised Linear Models (GLM) to investigate the effect of biotic and abiotic factors on VOC emissions. For this purpose, the 21 volatile compounds with higher contributions to the first six components selected through the PCA were used (Figure S1). The GLMs showed a significant effect of environmental variables on emissions of 14 volatile compounds, mostly fatty acid derivatives and terpenoids. However, the emissions of some compounds ((*Z*)-3-hexenol, (*Z*)-3-hexenyl acetate, (*Z*)-β-ocimene, (*E*)-β-caryophellene, γ-elemene, copaene and humulene) were not significantly affected by the factors tested in this study (Table S4).

Using K as a proxy for other nutrients, we found soil nutrients to be the main factor contributing to the differences in VOC emission of heather between sites. They significantly affected the emissions of the fatty acid derivatives (*Z*)-2-hexenol (*X*^2^ = 51.00, *df* = 1, *p* < 0.001), (*Z*)-3-hexenyl 2-methylbutyrate (*X*^2^ = 4.93, *df* = 1, *p* = 0.026), (*Z*)-3-hexenyl benzoate (*X*^2^ = 50.10, *df* = 1, *p* < 0.001), (*Z*)-3-hexenyl butyrate (*X*^2^ = 5.45, *df* = 1, *p* = 0.020), (*Z*)-3-hexenyl hexanoate (*X*^2^ = 10.20, *df* = 1, *p* = 0.001), (*Z*)-3-hexenyl isobutyrate (*X*^2^ = 7.23, *df* = 1, *p* = 0.007) and (*Z*)-3-hexenyl valerate (*X*^2^ = 8.06, *df* = 1, *p* = 0.005). In addition, the emissions of terpenoids (*E*,*E*)-α-farnesene (*X*^2^ = 15.10, *df* = 1, *p* < 0.001), α-gurjunene (*X*^2^ = 10.10, *df* = 1, *p* = 0.002), germacrene B (*X*^2^ = 5.79, *df* = 1, *p* = 0.016), germacrene D (*X*^2^ = 6.08, *df* = 1, *p* = 0.014), (*E*)-β-farnesene (*X*^2^ = 8.37, *df* = 1, *p* = 0.004) and (*E*)-DMNT (*X*^2^ = 39.30, *df* = 1, *p* < 0.001) were significantly affected by soil nutrients (Table S4).

Temperature was the second most important factor that explained the differences in the VOC emissions of this species. Among the compounds selected through PCA, temperature significantly affected (*Z*)-2-hexenol (GLM; *X*^2^ = 7.97, *df* = 1, *p* = 0.005), (*Z*)-3-hexenyl benzoate (GLM; *X*^2^ = 10.90, *df* = 1, *p* < 0.001), germacrene D (GLM; *X*^2^ = 21.10, *df* = 1, *p* < 0.001) and (*E*)-DMNT (GLM; *X*^2^ = 5.44, *df* = 1, *p* = 0.020). The effect of herbivory on VOC emissions by heather was minimal, only significant for hexyl acetate (GLM; *X*^2^ = 12.50, *df* = 1, *p* < 0.001) and germacrene B (GLM; *X*^2^ = 4.73, *df* = 1, *p* < 0.001), while soil water content had no significant impact on the volatile emissions of heather (Table S4).

## 3. Discussion

In this study, we show that total volatile emissions by heather are highly variable ranging from 2.61 ng gDW^−1^h^−1^ to 110.985 ng gDW^−1^h^−1^ for the lowest and highest emitting plant in its invaded range in New Zealand even within the same season (summer). This species emits large amounts of terpenoids (Table S2), which is consistent with a previous report in a temperate heath ecosystem in its native range [27]. Low levels of soil nutrients appear to be particularly important in regulating the VOC emissions of heather in this habitat. The soils with lowest levels of most nutrients (site 4)—in particular, potassium (K)—had significantly lower emissions of most fatty acid derivatives, some sesquiterpenes and the homoterpene (*E*)-DMNT (Table S2).

The effect of soil nutrients on VOC emission by plants is still poorly documented, but previous reports suggest that nutrient depletion has a negative impact on plant volatile emissions [29,30,31]. The role of K has been less explored than that of other nutrients (N and P), but this macronutrient plays a key role in stomatal conductance, enzyme activity, and plant responses to a wide range of biotic and abiotic stress [32], which may all impact VOC emissions. In addition, some evidence suggests that high levels of soil K can affect the production of secondary metabolites in plants causing increased production of both non-volatile (such as phenolics, flavonoids and ascorbic acid) and volatile compounds [33,34].

The production of constitutive defence compounds and the inducibility of such compounds can also be controlled by nutrient availability, with some plants investing more in their production at the expense of growth under nutrient limited conditions [35]. This exemplifies the dilemma faced by plants on whether to grow or defend in response to nutrient availability, which could be vital in determining the outcome of interactions between competing plants. Further studies are required to elucidate the role of individual nutrients, as we only used potassium as a proxy for other nutrients.

The main compounds with reduced emissions at site 4, which had nutrient-poor soil, were (*Z*)-3-hexenyl acetate, (*E*)-β-caryophellene and γ-elemene (Table S2). Fatty acid derivatives including (*Z*)-3-hexenyl acetate are typically involved in direct and indirect defence by repelling herbivores and attracting their natural enemies [36,37,38]. Terpenoids, on the other hand, represent the largest and most diverse class of plant secondary metabolites including VOCs [39]. A single plant organ can produce multiple terpenes, which makes it difficult to assign specific roles to individual compounds in this chemical class [40]. Despite that, available literature suggests that ecological roles of terpenes include direct and indirect defence against herbivores and pathogens, attraction of pollinators and protection against abiotic stress [11,41,42].

Herbivory is a well-known factor affecting VOC emissions, with green leaf volatiles (fatty acid derivatives) comprising about 50% of the VOCs released by plants attacked by chewing herbivores [11,13,43]. In our study, herbivory had a strong positive effect on the emission of hexyl acetate, a green leaf volatile [44,45], but negatively affected the release of germacrene B. The minimal effect of herbivore damage on VOC emissions in this study could be due to low herbivory on heather by native insects in New Zealand, and because of the lack of specialist herbivores at the selected sites. Further studies should explore the effect of the introduced biocontrol agent *Lochmaea suturalis* on the VOC emissions by heather.

Feeding damage observed on heather was caused by generalist species, and the slightly lower damage at the heather dominant site (site 1) and site 4, where another invasive plant (Scotch broom) is present, suggests that herbivorous arthropod communities are depauperate at sites where invasive plants are dominant. A recent review found that invasive plant species are often associated with an overall reduction in arthropod abundance and taxonomic richness [46]. However, changes in vegetation structure and high availability of litter and decaying vegetation caused by invasive plants can increase predators, detritivores and fungivores [46]. This agrees with the high number of spiders, oribatid mites, and fungus beetles (Cryptophagidae) found in this study (Figure 5). Most of the Hemiptera found on heather at site 4 were broom psyllids which were introduced into New Zealand in 1993 as a biocontrol agent for broom [47].

Elevated temperature is also known to increase the emission of various volatile compounds ranging from fatty acid derivatives to terpenoids [48,49,50], and these temperature-dependent emissions can be on both *de novo* synthesized and stored volatiles [51]. In this study, the emissions of (*Z*)-3- hexenyl benzoate, germacrene D and (*E*)-DMNT by heather were affected by temperature differences between sites. Temperature did not account for the emissions of other volatile compounds, which is likely due to the homogeneous weather during the VOC collection periods, indicating that there must be some stability in VOC emission that can withstand certain levels of environmental variation [52].

The effect of water stress on VOC emissions is not consistent in the literature and has been proposed to be dependent on plant species, duration and severity of water stress, as seen in isoprene-emitting species [51,53]. In our study, the differences in soil water content between sites did not have a significant effect on the variability of the identified VOCs. Although there were differences between some of our study sites, it is clear that heather is a highly adaptable species, growing in conditions ranging from well-drained soils to bogs [54,55], and is therefore not likely to be sensitive to minor fluctuations in soil water content.

A recent study found significant variation in the VOC emission of heather in response to experimentally induced elevated CO_2_, drought and night-time warming over six years [27]. The results show decreased monoterpene emissions up to 40% in response to elevated CO_2_. Experimentally induced drought also had a negative impact on monoterpene emissions immediately after treatment application and in the late growing season, while experimental night-time warming increased total emissions, showing the potential impact of climate change on heather VOC emissions [27]. In contrast, monoterpenes did not appear to be particularly affected by the biotic and abiotic variables measured in our study; this suggests that plants respond differently to natural variation in their environment than to severe or long-term stressors.

## 4. Conclusions

This work explores the chemical behaviour of the highly invasive environmental weed *Calluna vulgaris* at four different sites on the Central North Plateau of New Zealand. Our study provides the first evidence suggesting that volatile emissions of *C. vulgaris* are influenced by different environmental factors, with soil nutrients (K) being a major contributor to the variation in emissions under natural conditions in its invasive range. 

As this study was conducted under natural conditions, we acknowledge the possibility that variability in VOC emissions could also be linked to other variables not identified in our study. We found a common fungal volatile (1-octen-3-ol) in all sites, suggesting a possible effect of interaction of the target plants with microorganisms [28]. Furthermore, previous studies suggest that the composition of plant communities can have a strong effect on VOC emissions [56,57,58]. In this study, the site with lower volatile emissions had a strong presence of another invasive species, Scotch broom (*Cytisus scoparius*)*,* whereas the site with highest emissions was dominated by heather. The other two sites had a combination of heather with native species (i.e., either mānuka or *Dracophyllum*) evidencing differences in plant community composition (Table S1).

We therefore encourage further research to investigate the impact of plant-microbe interactions and other variables, such as neighbouring plant identity on heather volatile emissions. We also recommend exploring how these changes in a plant’s emissions influence the foraging behaviour of pollinators, soil arthropods, key herbivores and their natural enemies, as well as their impact on native plants. Such studies could provide valuable information on how volatiles contribute to the successful invasion of plants into novel environments.

## 5. Materials and Methods

### 5.1. Study Area

The study was conducted during the summer of 2017–2018, under natural conditions on the Central Plateau of the North Island, New Zealand. The region has a mean daily temperature of 12–13 °C in summer and 9–10 °C in winter, with low-fertility soils formed predominantly from volcanic ash [19,21,59]. Four sites (about 561 m^2^ per site) were set up: three within the Waiouru Military Training Area (WMTA) to the east of TNP and the fourth site near Erua, a small settlement on the western border of TNP (Table S1).

### 5.2. Sampling of Volatiles

Five plants of the same size and phenology were selected at each of the four sites. The introduced heather beetle (*Lochmaea suturalis*) has been released into this region as a bio control agent [26], but it is patchily distributed; therefore, we deliberately avoided sites where the beetle was present to reduce variation in herbivory within sites. Foliar volatile samples from heather were collected at each of the four sites. VOCs were sampled by using the “push–pull” headspace sampling technique [60]. Similar amounts of foliage from each sampled plant were enclosed in new multi-purpose cooking bags (AWZ Products, 50 cm × 30 cm) with their ends fastened. Using a portable PVAS22 pump (Volatile Assay Systems Rensselaer NY), carbon-filtered air was pushed into the bags through a PTFE tube (1.70 L/min) and simultaneously pulled out through another tube (1.20 L/min).

To collect the VOCs, a volatile collection trap with 30 mg HayeSep Q adsorbent (Volatile Assay Systems Rensselaer, NY, USA) was inserted in the pull tube [61,62]. Collections for each target plant were done for two hours during stable environmental conditions, over a period of four days in early December prior to heather flowering [63]. To control for collection time, random samples were collected simultaneously from different sites, and these were pooled for final analysis for each site. After VOC sampling, the foliage enclosed in the bags was excised and collected to measure herbivore damage. Plant material was subsequently oven dried at 60 °C for 72 h to estimate VOC emissions per dry weight (grams).

Collection filters were eluted using 200 μL of 95% hexane with 10 ng/mL nonyl acetate (C_11_H_22_O_2_) (Sigma Aldrich) as an internal standard. The samples were analysed using gas chromatography coupled to mass spectrometry (Shimadzu technologies) with a 30 m × 250 μm × 0.25 μm TG-5MS column and helium as a carrier gas. Operating conditions were as follows: injector temperature 230 °C; split ratio of 10; initial oven temperature at 50 °C, held for 3 min then increased to 95 °C at a rate of 5 °C/min. Tentative identification of compounds was achieved by comparing them with target spectra in the MS library from the National Institute of Standards and Technology (NIST) and, when available, verified by authentic standards (Sigma Aldrich).

### 5.3. Soil Sampling

To determine soil properties, four soil cores (15 cm deep × 3 cm diameter) were collected at random points surrounding each sampled plant, for a total of 20 soil cores per site. The fresh weight of each sample was measured on the day of collection, and then oven-dried (40 °C) to constant weight. Soil water content (SWC) was measured gravimetrically [64] and expressed as a percentage. After estimating SWC, cores collected from each site were homogenised and used for soil nutrient analysis (as averages for respective sites). Soil pH, Olsen phosphorus, potassium, calcium, magnesium, sodium, organic matter, total carbon and nitrogen were analysed by R. J. Hill Laboratories Limited, Hamilton–New Zealand.

### 5.4. Ambient and Soil Temperature Measurements

The ambient temperatures for experimental sites were obtained by installing temperature data loggers (Tinytag, Gemini) from mid-November to mid-December 2017. Soil temperatures were taken from five positions covering each site using a soil temperature probe.

### 5.5. Arthropods on Heather

Arthropods were collected from each sampled plant using the beating tray technique [65,66]. Beating of a branch on a tray was done immediately after volatile collection, but on an adjacent branch as the branch used for VOC collection was excised. Collected specimens were preserved in 70% ethanol and identified to order.

### 5.6. Herbivore Damage on Heather

The foliage enclosed in the bags from VOCs sampling was used to estimate herbivore damage. Due to the small size of the leaves, visible herbivore damage was assessed using a handheld magnifying glass. The number of damage marks seen on foliage was counted as illustrated in Figure 6a. To eliminate the bias of damage count being correlated with foliage size, the number of damage marks was divided by the dry weight (DW) of the respective foliage (i.e., herbivory per DW).

### 5.7. Data Analysis

Statistical analyses were performed using RStudio, Version 1.1.456 (RStudio: integrated development for R) [67]. The Shapiro–Wilk test was used to check the normality of herbivory, arthropod counts, and abiotic variable data; then, these were analysed using either analysis of variance (ANOVA) or a non-parametric Kruskal–Wallis test. When significant differences were found, Tukey’s honestly significant difference (Tukey’s HSD) or Mann–Whitney pairwise tests were used for post-hoc comparisons.

Principal component analysis (PCA) was performed using all the volatile compounds identified from the headspace of heather. PCA and descriptions of variable presentations in respective components were performed using the “FactoMiner” package [68]. A Generalised Linear Model (GLM) assuming Gamma distribution (log-link) was first performed to compare the proportions of VOC classes between the four sites using the GLM function in R. VOC classes were response variables while the four study sites were used as a categorical predictor. The relevel function was used to construct a set of level contrasts for the four sites [69,70] and the Wald test used to evaluate the significance of estimated effects [71].

A second GLM was then performed to determine the effect of environmental variables on volatiles emitted by heather. VOCs with higher contributions in PCA were the response variables. Herbivore damage, soil water content (SWC), average daytime temperature and primary macronutrients (nitrogen, phosphorus and potassium) were initially selected as potential predictor variables. These predictors were selected based on their importance to plant performance and VOC emissions [13,51,72,73]. To reduce collinearity, we performed a pairwise correlation between all predictor variables (Table S3), and those with high correlation were removed based on how they correlated with other variables [74]. This resulted in keeping only herbivory, ambient temperature, SWC, and potassium (as a proxy for nutrients other than nitrogen, which was strongly correlated with soil water content) in the final model, and all continuous predictor variables standardized prior to modelling [74]. In all the GLMs performed in this paper, we added a small constant (0.001) to all response variables to avoid the problem caused by expected values coming out as zero. This value was arbitrarily chosen but much smaller than the minimum observed emission rates for all the response variables and was tested for sensitivity to minimise the risk of contaminating findings while ensuring model convergence.

## Figures and Tables

**Table 1:** Comparison of soil properties between experimental sites. The medium or optimum range guidelines relate to Hills’ laboratories’ crop guides for mixed pasture.

Soil Properties	Site 1	Site 2	Site 3	Site 4	Reference (Medium Range)
Total nitrogen (%)	0.19	0.30	0.17	0.26	0.30–0.60
Total carbon (%)	3.70	6.00	3.10	2.80	NA
Olsen phosphorus (me/100 g)	5.00	3.00	4.00	3.00	20–30
Sodium (me/100 g)	0.06	0.06	0.10	<0.05	0.20–0.50
Magnesium (me/100 g)	0.48	0.31	0.34	0.16	1.00–1.60
Calcium (me/100 g)	2.70	1.60	1.50	0.70	4.0–10.0
Potassium (me/100 g)	0.18	0.24	0.22	0.13	0.40–0.60
Organic matter (%)	6.30	10.4	5.30	4.80	7.0–17.0
pH	5.70	5.70	5.50	5.80	5.8–6.2

NA = not applicable.

## Supplementary Materials

Figure S1 and Table S1 appear in the main text. The following are available online at https://www.mdpi.com/2223-7747/9/2/283/s1, Figure S1: Contribution of volatiles compounds in various principal components. Corrplot shows 15 components (Dim1 – Dim 15). The numbers in the graph indicate the following compounds; (1) hexyl acetate, (2) 1-hexanol, (3) (*Z*)-2-hexenol, (4) (*Z*)-3-hexenol, (5) (*Z*)-3-hexenyl 2-methylbutyrate, (6) (*Z*)-3-hexenyl acetate, (7) (*Z*)-3-hexenyl benzoate, (8) (*Z*)-3-hexenyl butyrate, (9) (*Z*)-3-hexenyl hexanoate, (10) (*Z*)-3-hexenyl isobutyrate, (11) (*Z*)-3-hexenyl valerate, (12) α-pinene, (13) α-terpineol, (14) β-myrcene, (15) β-pinene, (16) limonene, (17) linalool, (18) (*Z*)-β-ocimene, (19) (*E*,*E*)-α-farnesene, (20) α-gurjunene, (21) (*E*)-β-caryophellene, (22) δ-cadinene, (23) γ-elemene, (24) copaene, (25) germacrene B, (26) germacrene D, (27) humulene, (28) (*E*)-β-farnesene, (29) (*Z,E*)-α-farnesene, (30) (*E*)-DMNT, (31) 1-octen-3-ol, (32) decanal, (33) nonanal, Table S1: Geographical coordinates for study sites, Table S2: List of VOCs identified from the headspace of heather. Table showing means and standard deviation rate of emission based on square root transformed data, Table S3: Correlation test between predictor variables prior to performing GLM, Table S4: Summary of GLM (gamma distribution with log-link) based on VOCs with higher contribution in PC1 – PC6. Prior to modelling, a small constant 0.001 was added to all responses and the significance of predictor variables calculated using Wald test. Bold fonts with asterisks (*) indicate significant effect of predictors on response variables.
