# Supplementary material for: Natural Variation in Volatile Emissions of the Invasive Weed Calluna vulgaris in New Zealand
Source: Plants (Basel). 2020 Feb 21;9(2):283. doi: 10.3390/plants9020283 (PMC7076469; doi:10.3390/plants9020283)
Supplement: Supplementary file 1 [file plants-09-00283-s001.pdf]

Supplementary material

Table S1. Geographical coordinates for study sites

Site	Dominant woody species	Coordinates
Site 1	Heather	S39° 24.928' E175° 41.328'
Site 2	Heather and Mānuka	S39° 18.881' E175° 44.059'
Site 3	Heather – *Dracophyllum*	S39° 25.236' E175° 41.271'
Site 4	Heather – Broom	S39° 14.642' E175° 23.442'

Table S2. List of VOCs identified from the headspace of heather. Table showing means and standard deviation rate of emission based on square root transformed data

	Emission rate (mean ± SD)			
**Compound**	**Site 1**	**Site 2**	**Site 3**	**Site 4**
**Fatty acid derivatives**				
Hexyl acetate	0.37 ± 0.32	0.18 ± 0.22	0.12 ± 0.16	0.10 ± 0.13
1-hexanol	0.16 ± 0.10	0.08 ± 0.12	0.12 ± 0.11	0.03 ± 0.08
(Z)-2-hexenol	0.67 ± 0.41	0.47 ± 0.30	0.26 ± 0.29	ND
(Z)-3-hexenol	0.84 ± 0.64	1.20 ± 0.40	0.74 ± 0.55	0.86 ± 0.42
(Z)-3-hexenyl 2-methylbutyrate	1.00 ± 0.37	0.78 ± 0.37	0.50 ± 0.39	0.32 ± 0.15
(Z)-3-hexenyl acetate	4.80 ± 2.52	4.66 ± 1.74	3.84 ± 2.50	2.71 ± 1.13
(Z)-3-hexenyl benzoate	0.49 ± 0.12	0.29 ± 0.17	0.17 ± 0.12	ND
(Z)-3-hexenyl butyrate	2.56 ± 0.87	2.08 ± 0.83	1.27 ± 0.78	0.81 ± 0.43
(Z)-3-hexenyl hexanoate	0.21 ± 0.20	0.19 ± 0.19	0.05 ± 0.12	ND
(Z)-3-hexenyl isobutyrate	0.47 ± 0.13	0.36 ± 0.26	0.19 ± 0.25	0.08 ± 0.12
(Z)-3-hexenyl valerate	0.62 ± 0.13	0.51 ± 0.27	0.30 ± 0.11	0.10 ± 0.14
**Monoterpenoids**				
α-pinene	0.13 ± 0.17	0.22 ± 0.31	0.06 ± 0.09	0.25 ±
β-pinene	ND	0.29 ± 0.40	ND	0.18 ± 0.17
Linalool	0.14 ± 0.32	ND	0.20 ± 0.27	0.28 ± 0.29
α-terpineol	0.10 ± 0.22	ND	0.23 ± 0.21	0.11 ± 0.25
β-myrcene	0.18 ± 0.18	0.16 ± 0.23	0.20 ± 0.19	0.09 ± 0.19
Limonene	0.23 ± 0.15	0.19 ± 0.19	0.16 ± 0.15	0.07 ± 0.11
(Z)-β-ocimene	0.26 ± 0.25	0.60 ± 0.38	0.40 ± 0.25	0.26 ± 0.16

Table S2. Continued

**Sesquiterpenes**				
(*E,E*)-α-farnesene	1.10 ± 0.52	1.07 ± 0.32	0.96 ± 0.52	0.24 ± 0.18
α-gurjunene	0.21 ± 0.15	0.13 ± 0.13	0.15 ± 0.09	ND
(*E*)-β-caryophellene	0.51 ± 0.18	0.81 ± 0.51	0.32 ± 0.11	0.26 ± 0.17
γ-elemene	0.28 ± 0.18	0.27 ± 0.08	0.17 ± 0.12	0.10 ± 0.14
δ-cadinene	0.28 ± 0.18	0.21 ± 0.15	0.13 ± 0.09	0.12 ± 0.17
Copaene	0.07 ± 0.15	0.09 ± 0.19	ND	0.16 ± 0.16
Germacrene B	0.12 ± 0.12	0.05 ± 0.11	0.08 ± 0.11	ND
Germacrene D	0.72 ± 0.49	ND	0.09 ± 0.14	0.38 ± 0.38
(*E*)-β-famesene	0.18 ± 0.16	0.10 ± 0.14	0.13 ± 0.09	ND
Humulene	0.10 ± 0.10	0.19 ± 0.16	0.02 ± 0.05	0.03 ± 0.08
(*Z,E*)-α-farnesene	0.28 ± 0.63	0.65 ± 0.43	0.57 ± 0.52	ND
**Homoterpenes**				
(*E*)-DMNT	0.56 ± 0.35	0.33 ± 0.31	0.24 ± 0.20	ND
**Aldehydes**				
Decanal	0.33 ± 0.08	0.29 ± 0.07	0.27 ± 0.08	0.24 ± 0.10
Nonanal	0.31 ± 0.16	0.22 ± 0.15	0.22 ± 0.24	0.14 ± 0.21
**Other**				
1-octen-3-ol	0.80 ± 0.26	0.70 ± 0.33	0.50 ± 0.08	0.42 ± 0.57

*ND = not detected

Table S3. Correlation test between predictor variables prior to performing GLM

	Herbivory	SWC	Temperature	Nitrogen	Phosphorus	Potassium
Herbivory	0.1	0.2	-0.3	0.3	-0.2	0.3
SWC	0.2	1.0	-0.6	0.7	-0.6	-0.1
Temperature	-0.3	-0.6	1.0	-0.9	0.7	-0.4
Nitrogen	0.3	0.7	-0.9	1.0	-0.8	0.0
Phosphorus	-0.2	-0.6	0.7	-0.8	1.0	0.0
Potassium	0.3	-0.1	-0.4	0.0	0.0	1.0

Table S4. Summary of GLM (gamma distribution with log-link) based on VOCs with higher contribution in PC1 – PC6. Prior to modelling, a small constant 0.001 was added to all responses and the significance of predictor variables calculated using Wald test. Bold fonts with asterisks (*) indicate significant effect of predictors on response variables

Response	Predictor	B	CI		DF	X^2	*P*
			2.5%	97.5%			
**Hexyl acetate**	**Herbivory**	**1.232**	**0.549**	**1.914**	**1**	**12.500**	**< 0.001***
	Temperature	0.449	-0.568	1.466	1	0.749	0.387
	SWC	-0.488	-1.399	0.423	1	1.100	0.293
	Nutrients (K)	0.028	-0.745	0.800	1	0.005	0.944
**(Z)-2-hexenol**	Herbivory	-0.136	-0.823	0.551	1	0.150	0.699
	**Temperature**	**1.474**	**0.451**	**2.498**	1	**7.970**	**0.005***
	SWC	-0.195	-1.111	0.722	1	0.174	0.677
	**Nutrients (K)**	**2.833**	**2.055**	**3.610**	**1**	**51.000**	**< 0.001***
(Z)-3-hexenol	Herbivory	0.041	-0.579	0.661	1	0.017	0.896
	Temperature	0.218	-0.706	1.142	1	0.214	0.644
	SWC	0.419	-0.408	1.246	1	0.987	0.320
	Nutrients (K)	0.228	-0.473	0.930	1	0.407	0.524
**(Z)-3-hexenyl 2-methylbutyrate**	Herbivory	-0.042	-0.644	0.561	1	0.019	0.892
	Temperature	0.495	-0.403	1.393	1	1.170	0.280
	SWC	0.148	-0.656	0.952	1	0.131	0.717
	**Nutrients (K)**	**0.773**	**0.091**	**1.455**	**1**	**4.930**	**0.026***
(Z)-3-hexenyl acetate	Herbivory	0.287	-0.232	0.807	1	1.170	0.279
	Temperature	0.211	-0.563	0.985	1	0.284	0.594
	SWC	-0.047	-0.740	0.646	1	0.018	0.894
	Nutrients (K)	0.336	-0.252	0.923	1	1.250	0.263
**(Z)-3-hexenyl benzoate**	Herbivory	-0.134	-0.729	0.461	1	0.196	0.658
	**Temperature**	**1.494**	**0.607**	**2.380**	**1**	**10.900**	**< 0.001***
	SWC	0.143	-0.650	0.937	1	0.125	0.723
	**Nutrients (K)**	**2.434**	**1.760**	**3.107**	**1**	**50.100**	**< 0.001***
**(Z)-3-hexenyl butyrate**	Herbivory	-0.151	-0.726	0.424	1	0.266	0.606
	Temperature	0.418	-0.439	1.274	1	0.914	0.339
	SWC	0.149	-0.618	0.915	1	0.144	0.704
	**Nutrients (K)**	**0.775**	**0.124**	**1.425**	**1**	**5.450**	**0.020***
**(Z)-3-hexenyl hexanoate**	Herbivory	-0.718	-1.518	0.082	1	3.100	0.078
	Temperature	0.081	-1.111	1.273	1	0.018	0.894
	SWC	-0.441	-1.508	0.626	1	0.656	0.418
	**Nutrients (K)**	**1.479**	**0.573**	**2.384**	**1**	**10.200**	**0.001***
**(Z)-3-hexenyl isobutyrate**	Herbivory	-0.473	-1.098	0.151	1	2.200	0.138
	Temperature	0.256	-0.675	1.187	1	0.290	0.590
	SWC	-0.041	-0.874	0.793	1	0.009	0.924
	**Nutrients (K)**	**0.970**	**0.263**	**1.677**	1	**7.230**	**0.007***
**(Z)-3-hexenyl valerate**	Herbivory	-0.102	-0.681	0.477	1	0.119	0.730
	Temperature	0.349	-0.514	1.212	1	0.628	0.428
	SWC	-0.034	-0.806	0.739	1	0.007	0.932
	**Nutrients (K)**	**0.950**	**0.294**	**1.605**	**1**	**8.060**	**0.005***
(Z)-β-ocimene	Herbivory	-0.074	-0.627	0.478	1	0.070	0.792
	Temperature	-0.153	-0.977	0.670	1	0.133	0.716

	SWC	0.214	-0.523	0.952	1	0.325	0.569
	Nutrients (K)	0.588	-0.037	1.214	1	3.400	0.065
**(*E*,*E*)-α-farnesene**	Herbivory	-0.243	-0.773	0.287	1	0.809	0.368
	Temperature	0.393	-0.396	1.182	1	0.951	0.329
	SWC	-0.085	-0.792	0.621	1	0.056	0.813
	**Nutrients (K)**	**1.187**	**0.588**	**1.786**	**1**	**15.100**	**< 0.001***
**α-gurjunene**	Herbivory	-0.156	-0.839	0.527	1	0.200	0.654
	Temperature	0.410	-0.607	1.428	1	0.625	0.429
	SWC	-0.695	-1.606	0.215	1	2.240	0.135
	**Nutrients (K)**	**1.252**	**0.479**	**2.025**	**1**	**10.100**	**0.002***
(*E*)-β-caryophellene	Herbivory	0.196	-0.319	0.711	1	0.556	0.456
	Temperature	-0.475	-1.242	0.292	1	1.470	0.225
	SWC	-0.203	-0.890	0.484	1	0.336	0.562
	Nutrients (K)	0.291	-0.292	0.873	1	0.955	0.329
γ-elemene	Herbivory	0.098	-0.435	0.631	1	0.130	0.718
	Temperature	0.201	-0.593	0.995	1	0.247	0.619
	SWC	0.047	-0.663	0.758	1	0.017	0.896
	Nutrients (K)	0.388	-0.215	0.991	1	1.590	0.207
Copaene	Herbivory	1.564	-0.254	3.383	1	2.840	0.092
	Temperature	0.487	-2.223	3.197	1	0.124	0.725
	SWC	0.200	-2.226	2.626	1	0.026	0.872
	Nutrients (K)	-1.111	-3.169	0.948	1	1.120	0.290
**Germacrene B**	**Herbivory**	**-0.803**	**-1.526**	**-0.080**	**1**	**4.730**	**0.030***
	Temperature	0.419	-0.659	1.497	1	0.581	0.446
	SWC	-0.259	-1.223	0.706	1	0.276	0.599
	**Nutrients (K)**	**1.005**	**0.187**	**1.824**	**1**	**5.790**	**0.016***
**Germacrene D**	Herbivory	0.061	-0.589	0.710	1	0.034	0.855
	**Temperature**	**2.267**	**1.299**	**3.235**	**1**	**21.100**	**< 0.001***
	SWC	0.493	-0.373	1.360	1	1.240	0.265
	**Nutrients (K)**	**-0.925**	**-1.660**	**-0.190**	**1**	**6.080**	**0.014***
Humulene	Herbivory	-0.330	-1.165	0.505	1	0.599	0.439
	Temperature	-0.448	-1.693	0.797	1	0.498	0.480
	SWC	0.411	-0.704	1.525	1	0.522	0.470
	Nutrients (K)	0.557	-0.388	1.503	1	1.330	0.248
**(*E*)-β-farnesene**	Herbivory	-0.550	-1.298	0.199	1	2.070	0.150
	Temperature	0.436	-0.679	1.551	1	0.587	0.443
	SWC	-0.469	-1.468	0.529	1	0.850	0.357
	**Nutrients (K)**	**1.250**	**0.403**	**2.097**	**1**	**8.370**	**0.004***
**(*E*)-DMNT**	Herbivory	-0.468	-1.178	0.242	1	1.670	0.196
	**Temperature**	**1.259**	**0.201**	**2.317**	**1**	**5.440**	**0.020***
	SWC	-0.216	-1.164	0.731	1	0.201	0.654
	**Nutrients (K)**	**2.570**	**1.766**	**3.374**	**1**	**39.300**	**< 0.001***

* Estimated coefficient (β)
* Confidence interval (CI)
* Degree of freedom (DF)
* Potassium (K)
* Soil water content (SWC)

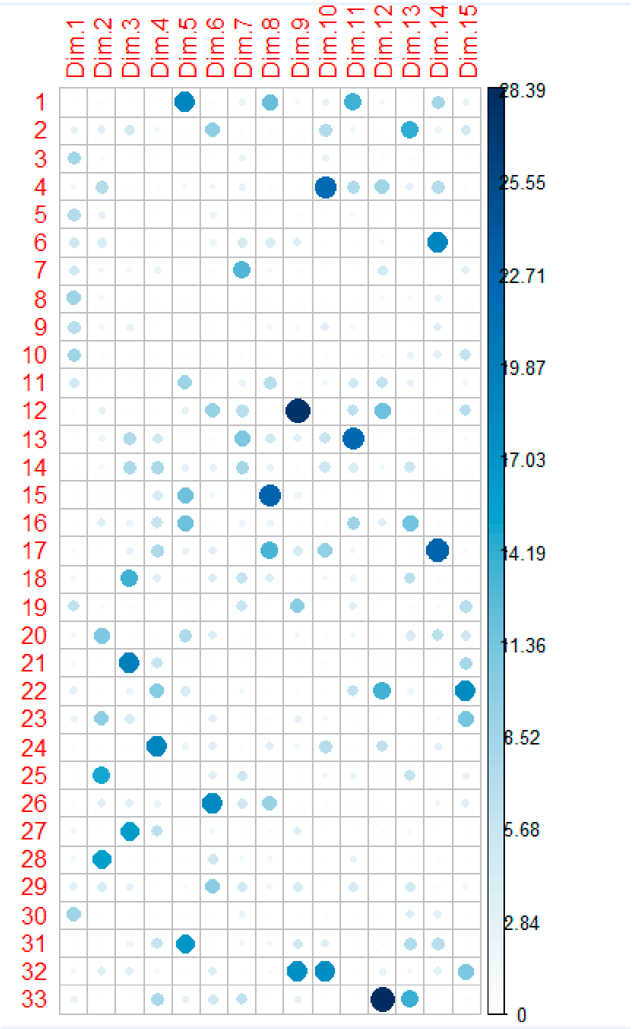

Figure S1. Contribution of volatiles compounds in various principal components. Corrplot shows 15 components (Dim1 – Dim 15). The numbers in the graph indicate the following compounds; (1) Hexyl acetate, (2) 1-hexanol, (3) (*Z*)-2-hexenol, (4) (*Z*)-3-hexenol, (5) (*Z*)-3-hexenyl 2-methylbutyrate, (6) (*Z*)-3-hexenyl acetate, (7) (*Z*)-3-hexenyl benzoate, (8) (*Z*)-3-hexenyl butyrate, (9) (*Z*)-3-hexenyl hexanoate, (10) (*Z*)-3-hexenyl isobutyrate, (11) (*Z*)-3-hexenyl valerate, (12) *α*-pinene, (13) *α*-terpineol, (14) β-myrcene, (15) β-pinene, (16) Limonene, (17) Linalool, (18) (*Z*)-β-ocimene, (19) (*E*,*E*)-*α*-farnesene, (20) *α*-gurjunene, (21) (*E*)-β-caryophellene, (22) δ-cadinene, (23) γ-elemene, (24) Copaene, (25) Germacrene B, (26) Germacrene D, (27) Humulene, (28) (*E*)-β-farnesene, (29) (*Z*,*E*)-*α*-farnesene, (30) (*E*)-DMNT, (31) 1-octen-3-ol, (32) Decanal, (33) Nonanal.
